# Obstructive Sleep Apnea Syndrome; a neglected cause of traffic collision among Iranian public transport drivers

**DOI:** 10.5249/jivr.v6i2.577

**Published:** 2014-07

**Authors:** Habibolah Khazaie, Azad Maroufi

**Affiliations:** ^*a*^Sleep Disorders Research Center, Kermanshah University of Medical Sciences (KUMS), Kermanshah, Iran.; ^*b*^Kurdistan University of Medical Sciences, Sanandaj, Iran.

Traffic collision ranks as the second most common cause of mortality among Iranian people.^1^ Several studies have reported that Obstructive Sleep Apnea Syndrome (OSAS) increases the risk of car crash. ^1,2,3^

About 27% of the Iranian population suffers from OSAS.^4^ Some studies have revealed that a history of witnessed apnea is the most important predictor of motor vehicle crash.^1^ In this report, we wish to present the results of our preliminary findings at the Sleep Disorders Research Center of Kermanshah University of Medical Sciences with regard to this problem among public transport drivers in western Iran. First of all, we screened 170 public transport drivers for objective OSAS using a Berlin Questionnaire. ^5^ Among these, 50 cases (29.5%) were distinguished as high risk for OSAS. In the next phase of the study, we evaluated these 50 cases using Actigraphy or Polysomnography. Results showed that 85% of them suffered from moderate to severe OSAS (AHI or RDI >15). Nearly all (95%) of them had daily sleepiness. Despite adequate education and advice about the necessity of treatment using CPAP, BiPAP or Auto CPAP; only two patients (4.5%) agreed to use Auto CPAP. Both of them showed poor compliance in their use of Auto CPAP and discontinued the treatment after only a few nights when followed up.

These preliminary results seem to indicate that most people with untreated OSAS in our country still continue to work in high-risk jobs, such as driving public transport vehicles. This risks not only their own lives but also those of other road-users and contributes to making Iranian roads among the most dangerous in the world.^6^

Future studies must focus on the prevalence of this syndrome among the population in general and among public transport drivers and others doing high-risk jobs in Iran in particular.

## References

[1] Montazeri A (2004). Road_traffic_related mortality in Iran:a descriptive study. Public Health.

[2] Pizza F, Contardi S, Mondini S, Cirignotta F (2012). Impact of sleep deprivation and obstructive sleep apnea syndrome on daytime vigilance and driving performance: a laboratory perspective. G Ital Med Lav Ergon.

[3] Birleanu LA, Rusu G, Mihaescu T (2010). Obstructive sleep apnea(OSA) syndrome and traffic accidents. Rev Med Chir Soc Med Nat Iasi.

[4] Khazaie H, Najafi F, Rezaie L, Tahmasian M, Sepehry AA, Herth FJ (2011). Prevalence of symptoms and risk of obstructive sleep apnea syndrome in the general population. Arch Iran Med.

[5] Abrishami A, Khajehdehi A, Chung F (2010). A systematic review of screening questionnaires for obstructive sleep apnea. Can J Anaesth.

[6] Drake C, Roehrs T, Breslau N, Johnson E, Jefferson C, Scofield H (2010). The 10-year risk of verified motor vehicle crashes in relation to physiologic sleepiness. Sleep.

